# Treadmill exercise ameliorates atherogenesis and vascular inflammation in ApoE^−/−^ mice via circulating exosome-derived let-7c-5p

**DOI:** 10.1038/s41598-025-30174-3

**Published:** 2025-12-09

**Authors:** Wenhuang Guo, Jinyun Wang, Zaoshang Chang, Shuo Lin, Guangyuan Sha, Shen Wang, Junhao Huang, Min Hu, Jingbo Xia

**Affiliations:** 1 Guangdong Provincial Key Laboratory of Physical Activity and Health Promotion, Guangzhou Sport University, Guangzhou, 510500 China; 2 Department of Physiology, Shaoyang University, Shaoyang, China

**Keywords:** Treadmill exercise, Atherosclerosis, let-7c-5p, Timp-3, Vascular inflammation, Atherosclerosis, miRNAs

## Abstract

Regular exercise training has been shown to significantly decrease atherosclerosis (AS) related mortality and hospitalization rates. Recent research has identified that circulating exosome-derived microRNAs (miRNAs) are closely related to the progression of AS through intercellular communication. But the role of exosome-derived miRNAs in exercise-mediated protection remains to be explored. This study proposes that exercise may ameliorate vascular dysfunction and plaque formation associated with AS by modulating the expression profile of exosomal miRNAs. In this study, ApoE^−/−^ mice were used and subjected to a ten-week treadmill exercise regimen to elucidate the molecular mechanisms by which exercise influences AS, specifically through alterations in exosomal miRNAs. The results demonstrated that exercise significantly diminished plaque area and enhanced both vascular endothelium-dependent vasodilation and cardiac function in AS mice. However, no significant differences in arterial mechanical stiffness were observed. Isolation and sequencing of serum exosomes revealed a marked decrease in serum let-7c-5p levels in AS mice after exercise. Correspondingly, let-7c-5p expression in arterial vessels was significantly reduced. The negative regulatory relationship between let-7c-5p and its target gene, tissue inhibitor of metalloproteinases-3 (Timp-3), was subsequently validated through dual luciferase assays. Concurrently, an increase in Timp-3 expression in arterial vessels was observed, alongside a significant reduction in the inflammatory factors tumor necrosis factor-α (TNF-α), interleukin-6 (IL-6), and matrix metalloproteinase-9 (MMP-9). Cellular experiments further corroborated the regulatory effect of let-7c-5p on Timp-3. Overall, these results suggest that exercise promotes Timp-3 expression by downregulating let-7c-5p carried by circulating exosomes, thereby mitigating atherogenesis and vascular inflammation in ApoE^−/−^ mice.

**Supplementary Information:**

The online version contains supplementary material available at 10.1038/s41598-025-30174-3.

## Introduction

Atherosclerosis (AS), a complex chronic inflammatory disease, is characterized by lipid deposition in arterial walls that leads to hardening and narrowing of arteries. AS has been recognized as a major contributor to cardiovascular diseases, which is the leading cause of death around the world^1^. AS affects cardiovascular health through multiple mechanisms including endothelial dysfunction^2^, vascular smooth muscle cell (VSMC) proliferation^3^, inflammatory responses^4^, and vascular calcification^5^. The accumulation of atheromas, or atherosclerotic plaques, gradually obstructs blood flow, causes arteries to harden, and may result in heart attacks or strokes^6^. Although we have made great strides in comprehending the mechanisms that lead to AS and have seen a reduction in cardiovascular deaths, the overall disease burden is still considerably high^7^. Consequently, the development of novel therapeutic strategies is still imminent. Cardiovascular diseases can be prevented or treated with lifestyle changes, including a nutritious diet, regular exercise, proper sleep, limited alcohol use, and not using tobacco^8^. Among these, exercise training can not only directly provide endogenous heart protection for AS^9^, but also reduce the incidence rate and mortality of AS^10^. However, the mechanism behind the protective effect of exercise training on AS is not completely understood.

MicroRNAs (miRNAs), which are small RNA molecules around 22 nucleotides in length and do not code for proteins, are chiefly responsible for the regulation of gene expression after transcription^11^. MiRNAs are gaining widespread attention for their physiological roles in the cardiovascular system and are key molecules in maintaining cardiovascular homeostasis. Their functions include regulating heart failure, cardiac fibrosis, vascular remodeling, AS, and lipid metabolism, among others^12–15^. Recent studies have demonstrated that miRNAs carried by exosomes play a significant role in the development of AS. Exosomes, nanoscale extracellular vesicles, can transport various biomolecules including proteins, lipids, mRNA, and miRNAs. These vesicles influence the functions of recipient cells through intercellular communication mechanisms^16^.

MiRNAs play a crucial role in the instability and rupture of atherosclerotic plaques. Studies have shown that miRNAs can be transmitted between cells through exosomes, participating in regulating the formation, development, and rupture processes of atherosclerotic plaques^17^. Furthermore, miRNAs carried by exosomes can influence the progression of AS through mechanisms such as regulating inflammatory responses and apoptosis. These miRNAs include miR-126, miR-146a, miR-155^18^, and miR-492^19^, which may play an important role in the protection of AS by exercise. However, the mechanism of these miRNAs in exercise mediated AS protection is still unclear. In addition, miRNAs, as potential therapeutic targets, may provide valuable clues for finding ways to improve or treat AS.

In this study, we identified let-7c-5p as a key factor that mediates the beneficial effects of exercise and delays the progression of AS. Inhibition of let-7c-5p was observed to increase the expression of Timp-3 and decrease the levels of inflammatory factors. These findings elucidate a novel endogenous protective mechanism that exercise-downregulated let-7c-5p confers protection against atherogenesis and vascular inflammation in AS mice.

## Materials and methods

### Animals

The study was approved by the Animal Experimental Ethics Inspection of Guangzhou Sport University (protocol code 2024DWLL-36). All methods were performed in accordance with the relevant guidelines and regulations, and followed the ARRIVE guidelines. A total of 39 male C57BL/6 J mice aged 7 weeks were purchased from Guangdong Medical Laboratory Animal Center, of which 26 were ApoE knockout mice (ApoE^−/−^ Mice), and 13 were wild-type (WT) C57BL/6 J mice. These mice were housed in a specific pathogen free laboratory animal facility under standardized conditions, including a temperature of 24 ± 2 °C, relative humidity of 50 ± 5%, and a 12-h light/dark cycle. The mice were allocated into three groups (n = 13 per group): WT mice on a standard diet, designated as the Control group; and ApoE^−/−^ mice on a high-fat, high-cholesterol diet, further subdivided into sedentary (AS-SED) and exercise (AS-EX) groups. Both the standard diet and the high-fat, high-cholesterol diet (40% kcal fat and 1.25% kcal cholesterol) were sourced from the Guangdong Medical Laboratory Animal Center. This dietary regimen was administered for a duration of 16 weeks. Body weight measurements were systematically recorded throughout the study duration. Following the experiments, the mice were euthanized via cervical dislocation under 2.5% isoflurane anesthesia. Mean body weights prior to euthanasia were 26.69 ± 1.51 g of the Control group, 26.61 ± 2.03 g of the AS-SED group, and 27.64 ± 2.96 g of the AS-EX group.

### Exercise protocol

During the experimental protocol, mice in the Control group and the AS-SED group were housed in cages with unrestricted activity. Animals from the AS-EX group were submitted to a treadmill exercise training program on an electric treadmill five consecutive days/week with a 0° incline for 10 weeks after 6-week-high-fat, high-cholesterol diet. The details of the intervention are as follows. The AS-EX group engaged in training five days per week, beginning with an initial week of adaptive exercise. During this period, the treadmill was set at a 0° incline, with running speeds progressively increased from 10 m per minute to 14 m per minute, and the duration of each session extended from 35 to 60 min. Animals were afforded a 2-min rest interval following every 15 min of exercise. The formal training regimen consisted of maintaining the treadmill at a 0° incline with a constant running speed of 14 m per minute, culminating in a total exercise duration of 60 min per session. Each session included 15 min of running followed by a 2-min rest period. This regimen was implemented five days per week, with two days allocated for rest, over the course of 10 weeks as previous study introduced^20^.

### Echocardiographic assessment

A high-resolution ultrasound imaging system (VINNO 6, VINNO Corporation, China) was used to perform transthoracic echocardiography in M-mode with a 23 MHz probe frequency. Left ventricular ejection fraction (EF), fractional shortening (FS), and cardiac output (CO) were calculated from the dimensions measured by echocardiography. As reported in the literature, pulse wave velocity (PWV) was employed to assess vascular stiffness^21^.

### Tissue samples collection

Sampling was performed 24 h following the final exercise session, following a 12-h fasting period. Mice were anesthetized using isoflurane, and blood samples were obtained from the retro-orbital venous plexus prior to euthanasia via cervical dislocation. The abdominal and thoracic cavities were immediately opened, and perfusion was performed with PBS through the left ventricle. Subsequently, the heart and aorta were excised and fixed overnight in 4% paraformaldehyde. Blood samples were transferred to 1.5 mL centrifuge tubes, allowed to clot for 30 min, and centrifuged at 3000 rpm at 4 °C for 15 min. Serum was collected and stored at -80 °C for subsequent experiments.

### Mechanical stiffness testing

Arterial elasticity and stiffness were evaluated using mechanical stiffness testing as previous described^22^. A preheated 37 °C Ca^2+^ and Mg^2+^-free PBS was added to a Multi Wire Myograph System chamber (Model 620 M, DMT, Denmark). A ~ 2.0 mm thoracic aorta ring, without perivascular adipose tissue, was placed in the chamber and stretched 1 mm every 3 min until failure. The elastic modulus was derived from the stress–strain curve, with strain (λ) = Δd/d (i) and stress (t) = F/2HD, where F is the load, H is wall thickness, D is vessel length, Δd is diameter change, and d (i) is initial diameter. Thoracic aortic diameter and wall thickness were determined by hematoxylin–eosin (H&E) staining (G1005, Servicebio, China), and vessel length was measured with a ruler. The elastin elastic modulus (EEM) and collagen elastic modulus (CEM) were calculated from the slopes of the stress–strain curves at the smooth and steep linear sections, respectively.

### Endothelial dependent aortic relaxation analysis

Aortic relaxation was assessed using acetylcholine (ACh, A6625, Sigma-Aldrich, USA)^23^. A 3 mm proximal thoracic aorta section was mounted on a force transducer in a Multi Wire Myograph System (Model 620 M, DMT, Denmark) with Krebs–Ringer solution (pH 7.4). The ring was gradually stretched to a predefined optimal tension (30 mN) and stabilized for 30 min. Subsequently, the tissue was preconstricted with 1 µmol/L phenylephrine (P1240000, Sigma-Aldrich) for 60–90 min. Endothelial function was evaluated by measuring relaxation responses to incremental concentrations of ACh. The concentration that leads to half-maximal inhibition in the Control group is known as IC50. IC50 values were changed to pIC50 to simplify calculations. The pIC50 values were -log IC50.

### Aortic lesion assessment

Oil Red O staining was employed to evaluate the development of aortic plaques. Three intact aortas, extending from the aortic arch to the iliac bifurcation, were collected from each experimental group and subjected to Oil Red staining (GP1077, Servicebio, China). The hearts were dehydrated using 30% sucrose and subsequently embedded in OCT compound (Takara, Japan). Continuous sections were then cut at a thickness of 6 μm using a cryostat. The aortic root sections were stained with Oil Red for lipid droplet accumulation examination, H&E (GP1077, Servicebio, China) for morphological examination and Masson’s trichrome (G1006, Servicebio, China) for the visualization of collagen fibers. Heart fibrosis was assessed with Sirius Red staining (Y026186, Beyotime Biotechnology, Shanghai, China). Dihydroethidium (DHE) staining (Thermo Fisher, USA) was performed as previously described^24^. Stained sections were imaged using a Leica microscope imaging system (DM4B, Leica, Germany), and the resulting images were analyzed with ImageJ software (NIH, USA).

### Isolation of exosomes

Due to the limited amount of serum from each mouse, the serum from 5 mice was pooled into one tube for exosome isolation. The isolation was conducted using a differential ultracentrifugation approach. Initially, the serum was centrifuged at 3000*g* for 10 min to remove cellular debris, followed by careful aspiration of the supernatant, which was then transferred to a fresh centrifuge tube. Subsequently, the supernatant was subjected to centrifugation at 10,000*g* for 30 min to eliminate larger vesicles. The resulting supernatant was then filtered through a 0.22 μm pore-size membrane. The filtrate was centrifuged at 100,000*g* for 1 h to pellet the exosome fraction, which appeared as a white precipitate at the bottom of the tube. This pellet was resuspended in PBS and centrifuged again at 4 °C and 100,000*g* for 1 h to further purify the exosomes. Finally, the exosome pellet was resuspended in 100–200 μL of PBS for subsequent analysis.

### Identification of exosomes

Further characterization of the isolated exosomes was performed using nanoparticle tracking analysis (NTA) with NanoSight software, transmission electron microscopy (TEM), and western blotting.

#### Nanoparticle tracking analysis (NTA)

NTA measured exosome size and concentration in liquid using light scattering and Brownian motion. The NanoSight NS300 (Malvern Instruments, UK) with a 405-nm laser detected nanovesicles. Five 60-s videos per sample were analyzed with NTA 3.0 software. The hydrodynamic diameter is calculated using the Stokes–Einstein equation: D = kT/6πηr, where D is the diffusion coefficient, k is Boltzmann’s constant, T is absolute temperature, r is particle radius, and η is fluid viscosity.

#### Transmission electron microscope (TEM)

Carefully dispense 20 μL of the exosome sample onto a copper grid and allow it to settle for 1 min. Subsequently, employ filter paper to remove any excess liquid from the grid’s surface. Following this, apply 20 μL of uranyl acetate to the copper grid, permit it to settle for 1 min, and again use filter paper to absorb the surplus liquid. Allow the copper grid to dry at room temperature for several minutes. Once the grid is dried, perform electron microscopy examination and imaging at an accelerating voltage of 100 kV. The imaging results were obtained using a transmission electron microscope (HT-7700, Hitachi, Japan).

### Western blotting

The extraction of proteins was performed using radio immunoprecipitation assay (RIPA) lysis buffer (P0013B, Beyotime Biotechnology, China) containing 12% protease and phosphatase inhibitors, followed by separation via sodium dodecyl sulfate polyacrylamide gel electrophoresis (SDS-PAGE). After transferring proteins to polyvinylidene difluoride membranes, the blots were treated with 5% nonfat dry milk dissolved in TBS + TWEEN 20 (TBS-T) for 60 min at room temperature. The membrane was incubated overnight at 4 °C in a mixture of prepared rabbit anti-TSG101 (1:1000, ab125011, Abcam, UK); rabbit anti-CD63 (1:1000, A19023, Abclonal, US); and rabbit anti-CD9 (1:1000, BM4212, BOSTER, China) primary antibodies. The membrane was then incubated for approximately 1 h at room temperature in a solution of goat anti-rabbit secondary antibody (1:5000, 31460, Invitrogen, US). After washing the membrane at room temperature for 10 min, the membrane was drained and an equal volume mixture of ECL A/B solution (1810212, Clinx, China) was added to the membrane and allowed to react in the dark for 5 min.

#### miRNA sequencing

For the purpose of miRNA sequencing, exosomes were isolated from the serum of mice in both the AS-SED and AS-EX groups. These exosomes were subsequently sequenced. The RNA sequencing and subsequent data analysis were conducted by Shanghai Biotechnology Co., Ltd.

#### RNA isolation and quantitative real-time polymerase chain reaction

Total RNA was extracted from cultured cells and the aorta using TRIzol Reagent (Invitrogen, USA). The extracted RNA was then converted into first-strand complementary DNA (cDNA) using the SweScript RT I First Strand cDNA Synthesis Kit (with gDNA Remover) (G3331-100, Servicebio, China). Quantitative Real-Time Polymerase Chain Reaction (qRT-PCR) was conducted employing the 2 × SYBR Green qRT-PCR Master Mix (High ROX) (G3322-15, Servicebio, China) in accordance with the manufacturer’s instructions. The fold-change for miRNA relative to U6 was determined by the formula 2^−ΔΔCt^. Primers for let-7 family were synthesized by Wuhan GeneCreate Biological Engineering Co., Ltd., whereas primers for IL-6, TNF-α, Timp-3, GAPDH, and MMP-9 were synthesized by Shanghai Generay Biotech Co., Ltd. The sequences of primers are detailed in Supplementary material.

#### Reporter plasmid construction and luciferase reporter assays

The wild-type (WT) and mutant (MT) Timp-3 constructs were designed according to specific mutation sites and synthesized by Wuhan GeneCreate Biological Engineering Co., Ltd. The luciferase reporter plasmids, Timp-3 WT 3′-untranslated region (3′-UTR) and Timp-3 MT 3′-UTR, were developed by Wuhan GeneCreate Biological Engineering Co., Ltd. These plasmids were co-transfected with either a Negative Control (NC) antagomir, let-7c-5p antagomir, NC mimic, or let-7c-5p mimic into 293T cells. Following a 48-h transfection period, the cells were harvested and lysed. Luciferase activity was subsequently measured using the Double-Luciferase Reporter Assay Kit (JKR23008, Genecreate, China) in conjunction with a chemiluminescence immunoanalyzer (BK-L96C, BIOSINO, China). Renilla luciferase served as an internal control, and the relative luciferase activity of the target reporter gene was determined by calculating the ratio of firefly luciferase to Renilla luciferase relative light units (RLU). The sequences of miRNAs are detailed in Supplementary material.

#### Immunohistochemistry and immunofluorescence staining

Sections of the brachiocephalic trunk and thoracic aorta were fixed with 4% PFA. For immunohistochemistry, rabbit anti-Timp-3 protein (1:100, PA5-32621, Thermo Fisher, US) was used to incubate the sections of the brachiocephalic trunk and thoracic aorta to determine the content of Timp-3. Subsequently, the sections were incubated with goat anti-rabbit secondary antibody (G1213-100UL, Servicebio, China). The content of Timp-3 in each lesion was evaluated as the percentage of positively stained area relative to the total lesion area. The same protocol was applied with CD68 (GB113109, Servicebio, China).

For immunofluorescence, mouse anti-TNF-α (1:100, 60291-1-Ig, Proteintech, China); mouse anti-IL-6 (1:300, 66146-1-Ig, Proteintech, China); and rabbit anti-MMP-9 (1:300, 10375-2-AP, Proteintech, China) primary antibodies were incubated overnight. MMP-9 was incubated with goat anti-rabbit secondary antibody (1:500, 8889S, Cell Signaling Technology, US) for 1 h. TNF-α and IL-6 were incubated with goat anti-mouse secondary antibody (1:500, 4408S, Cell Signaling Technology, US). The stained sections were photographed using a Leica microscope imaging system (DM4B, Leica, Germany), and the resulting images were analyzed with ImageJ software (NIH, USA).

#### Cell culture and treatment

The MOVAS (mouse aortic smooth muscle cells), sourced from SAINT-BIO (Shanghai, China), were cultured in high-glucose DMEM (G4524-500ML, Servicebio, China) with 10% fetal bovine serum (A3160901, Gibco, USA) and 1% penicillin/streptomycin(G4003-100ML, Servicebio, China), incubated at 37 °C in a humidified 5% CO_2_ atmosphere. Cells in optimal condition during the logarithmic growth phase were selected and subjected to digestion with 0.25% trypsin (G4011-100ML, Servicebio, China). Following centrifugation, the supernatant was removed, and the cells were re-suspended and inoculated into six-well plates for subsequent experimentation. The Timp-3 overexpression plasmid (pcDNA-Timp-3, NM_011595) and the pcDNA3.1 empty vector were synthesized by Hunan Fenghui Biotechnology Co., Ltd. To down-regulate Timp-3 expression, small interfering RNAs (siRNAs) targeting Timp-3 (si-Timp-3) were procured from Wuhan GeneCreate Biological Engineering Co., Ltd., with scrambled oligonucleotides (si-NC) serving as controls. The let-7c-5p mimic was utilized to simulate let-7c-5p activity, while the let-7c-5p inhibitor (anti-let-7c-5p) was employed to suppress let-7c-5p, with miR-NC and anti-miR-NC as respective controls. Transient transfection with plasmids or nucleotides was performed at 70% cell confluence using Lipofectamine 2000 (11668027, Invitrogen, US), followed by treatment with 50 mg/mL ox-LDL (oxidized low-density lipoprotein) (YB-002, Yiyuan Biotech, China) for 24 h. The siRNA transfection concentration was 150 pM, miRNA mimic transfection concentration was 200 pM, the miRNA inhibitor transfection concentration was 200 pM, and the plasmid transfection amount was 5 μg in 6-well plates. The sequences of siRNAs and miRNAs are detailed in Supplementary material. Cell viability analysis was performed by using the CCK-8 Cell Counting Kit (C0039, Beyotime Biotechnology, China). The intracellular ROS generation was evaluated by measuring the oxidation of 5-(and 6)-chloromethyl-2′7′-dichlorodihydrofluorescein diacetate (CM-H2DCFDA, C6827, Thermo Fisher, USA) with flow cytometry according to the manufacturer’s instructions, as previously described^24^. CM-H2DCFDA is a chloromethyl derivative of H2DCFDA, an indicator for ROS in cells. Intracellular ROS concentration was determined by the mean fluorescence intensity (MFI) of 10,000 cells.

#### Statistical analysis

Data analysis was performed with GraphPad Prism version 8.0 software. The outcomes are presented as the mean plus or minus standard error of the mean (Mean ± SEM). Among three groups, statistical analysis was performed using one-way ANOVA with Tukey’s multiple comparisons test. Comparisons between two groups were analyzed using unpaired and two-tailed Student’s t test. Values of *P* < 0.05 were considered statistically significant.

## Results

### Treadmill exercise improves AS and cardiac function

**Fig. 1:**
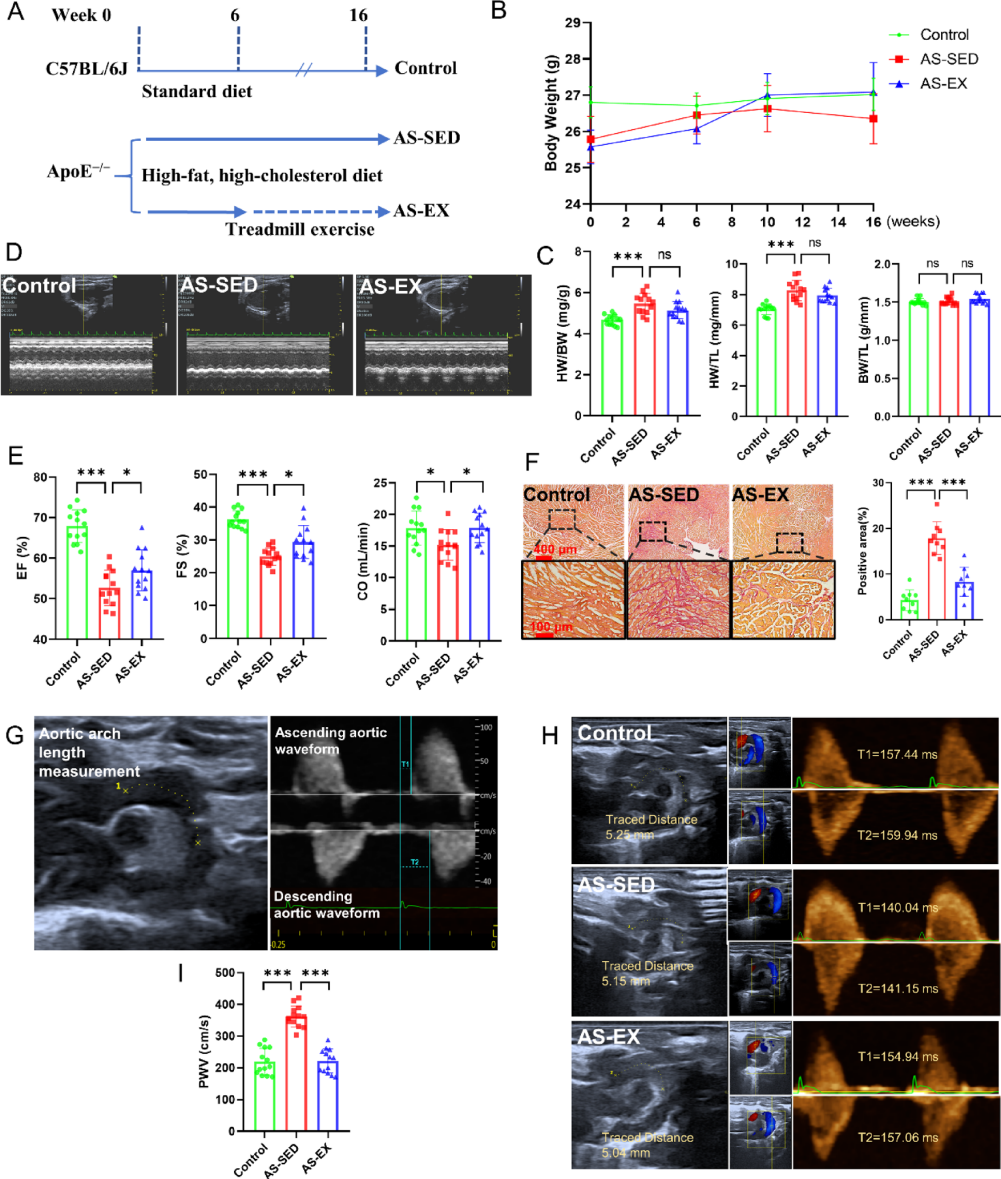
Exercise improves AS and cardiac function in ApoE^−/−^ mice. (**A**) The exercise and diet regimen. (**B**) Body weight change curve for 16 weeks. (**C**) The ratio of heart weight (HW) to body weight (BW), the ratio of heart weight (HW) to tibia length (TL), and the ratio of body weight (BW) to tibia length (TL) (n = 13 per group). (**D**) Cardiac function was evaluated using echocardiography. (**E**) Results of cardiac function ultrasound indexes include EF (ejection fraction), FS (fractional shortening) and CO (cardiac output (n = 13 per group). (**F**) Representative images and quantification of myocardium stained with Sirius Red for myocardial injury (n = 9 per group). (**G**) Selection of measurement area and calculation time for PWV (pulse wave velocity). Aortic arch PWV was determined as PWV = Distance/(T2–T1) cm/s. (**H**) Detection of PWV by echocardiography. (**I**) The bar graph represented the statistical results of the PWV values (n = 13 per group).*P < 0.05, **P < 0.01, ***P < 0.001, ns indicating *P* > 0.05.

Figure 1A shows the diet/exercise protocol. Figure 1B illustrated the changes in the body weights of the animals in different groups during the experiment. As shown in Fig. 1C, the heart weight (HW) to body weight (BW) ratio in the AS-SED group was notably higher than in the Control group. The ratio of heart weight (HW) to tibia length (TL) shows the same trend. In order to evaluate the cardiac function, left ventricular EF, FS, and CO were measured (Fig. 1D). The results demonstrated that compared to the Control group, the indicators of cardiac function, including EF, FS, and CO, decreased significantly in the AS-SED group, whereas compared with the AS-SED group, the indicators were significantly increased in the AS-EX group (Fig. 1E). In addition, the Sirius Red staining disclosed a massive fibrosis in AS-SED mice myocardium compared with Control mice, whereas the positive area was significantly decreased in the AS-EX mice compared with AS-SED mice (Fig. 1F). Previous studies indicated have that PWV can be used as an indicator of arterial stiffness, reflecting the degree of AS^25^. Thus, PWV was measured as an index of arterial stiffness (Fig. 1G, H). The results showed that, compared with the Control group, PWV was significantly elevated in the AS-SED group, whereas it was significantly decreased in the AS-EX group (Fig. 1I). Therefore, treadmill exercise can improve arterial stiffness and cardiac function in atherosclerotic mice.

### Assessment of arterial stiffness and endothelium-dependent vasodilation responses in isolated mouse aortas

**Fig. 2:**
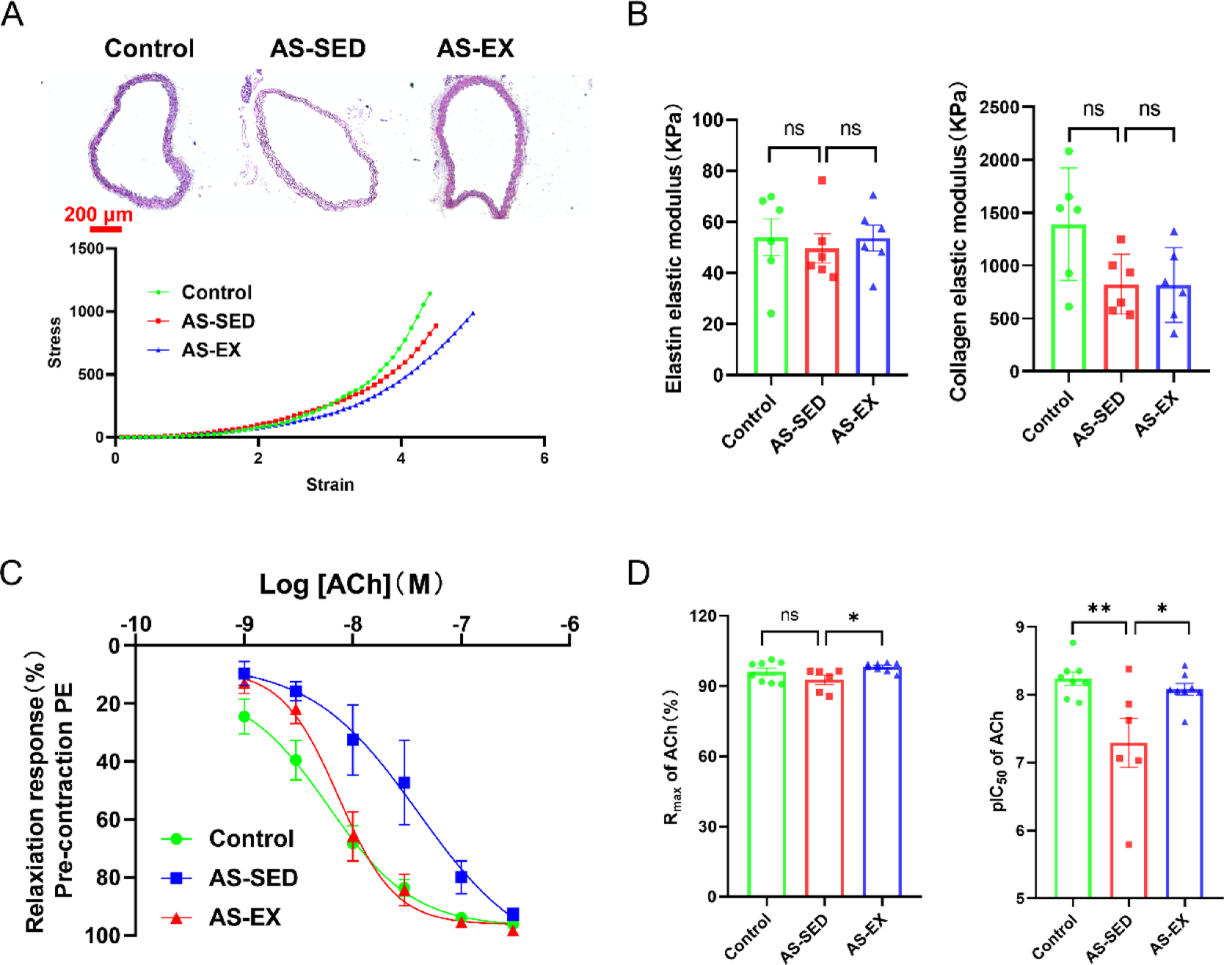
Assessment of arterial stiffness and endothelium-dependent vasodilation. (**A**) Representative H&E staining images of the proximal thoracic aorta from each group and the stress–strain curves. Strain (λ) = Δd/d (i) and stress (t) = F/2HD, where F is the load, H is wall thickness, D is vessel length, Δd is diameter change, and d (i) is initial diameter. (**B**) Quantification of the elastin elastic modulus (EEM) and collagen elastic modulus (CEM) of the proximal thoracic aorta from each group (n = 6 per group). (**C**) Concentration–response curves of mouse aortas to acetylcholine (ACh) endothelium-dependent vasodilator (n = 6–8 per group). PE, phenylephrine. (**D**) Results for maximum contraction (Rmax) induced by ACh and drug sensitivity (pIC50) to ACh. *P < 0.05, **P < 0.01, ns indicating *P* > 0.05.

Isolated mouse aortas were subjected to mechanical testing to assess stiffness. The stress–strain curves was shown in Fig. 2A. No significant differences were observed in the EEM among the three groups (Fig. 2B). Also, no significant difference in CEM was found among the three groups (Fig. 2B). Studies have found that short-term inactivity can lead to a decline in endothelium-dependent dilatation function in patients with coronary arteriosclerosis^26^. We next examined the effects of exercise intervention on ACh-induced endothelium-dependent relaxation. As shown in Fig. 2C, the endothelium-dependent vasodilator ACh produced a concentration-dependent relaxation of PE-induced tone in all arteries. As shown in Fig. 2D, maximum percentage of contraction in the AS-SED group was lower than that in the Control group though not significant. However, maximum percentage of contraction was significantly increased in the AS-EX group compared with the AS-SED group (Fig. 2D). Sensitivity to ACh was expressed as pIC50 value. The pIC50 value in the AS-SED group was lower than that in the Control group with a significant difference. The pIC50 value in the AS-EX group was higher than that in the AS-SED group (Fig. 2D). The AS-EX group with higher pIC50 values exhibited stronger endothelium-dependent vasodilation to ACh. These results indicated that exercise intervention markedly improved the vascular endothelial function in AS mice. However, it was unable to restore vascular endothelial function to its normal level.

### Treadmill exercise reduces atherosclerotic plaque formation

**Fig. 3:**
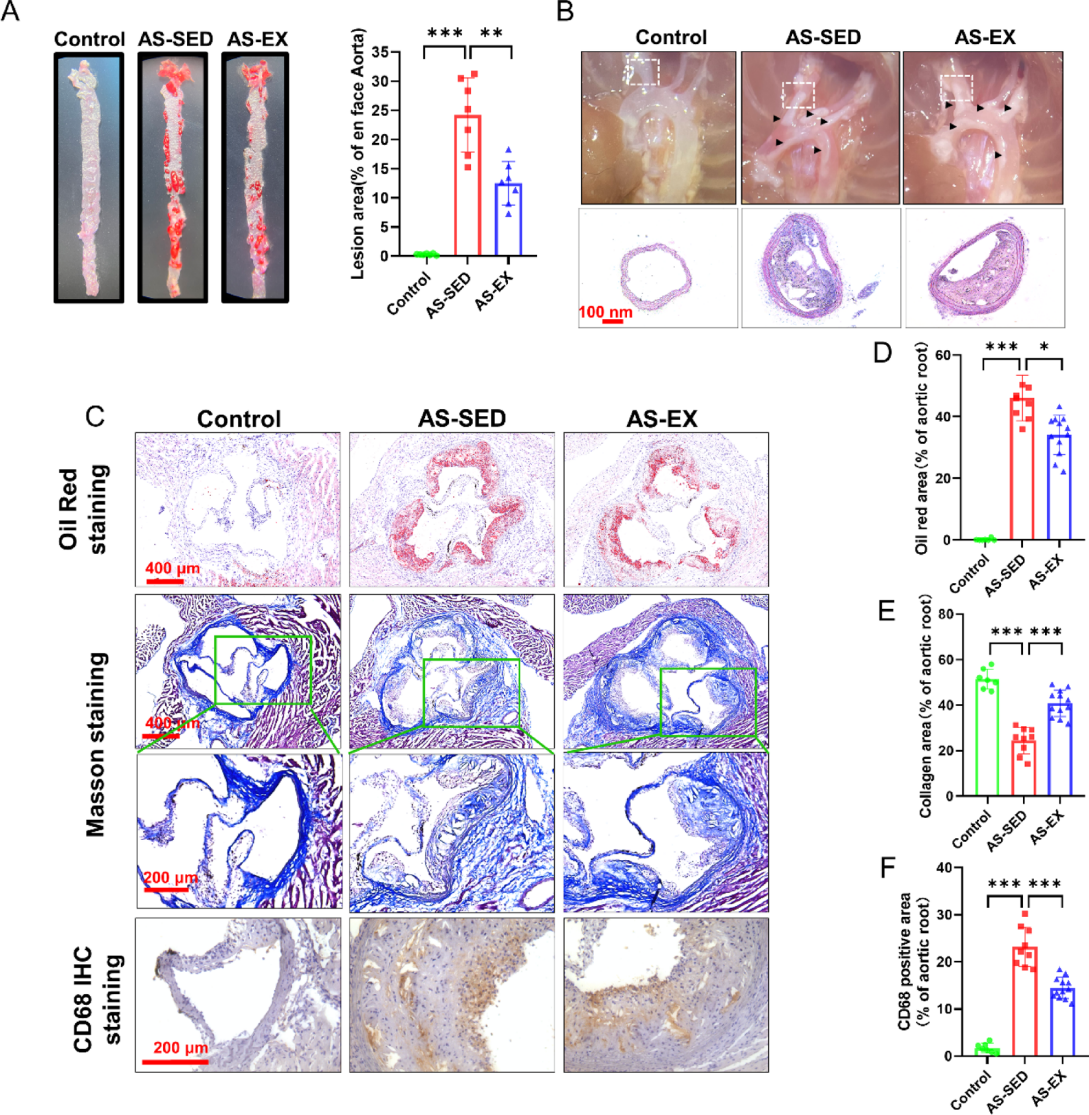
Exercise reduces aortic plaque formation. (**A**) Representatives of morphometric en face Oil Red O-stained lesions (red) in the aorta and statistical results (n = 7 per group). (**B**) The aortic arches were exposed, photographed and representative H&E staining images of the brachiocephalic arteries from three groups. (**C**) Representative images of Oil Red O staining, Masson staining and CD68 IHC staining in aortic root cross-sections. (**D**) Quantification of the Oil red area (n = 7–12 per group). (**E**) Quantification of collagen content (n = 7–12 per group). (**F**) Quantification of CD68 positive area (n = 7–12 per group). *P < 0.05,  *P < 0.01, ***P < 0.001.

Aortas were visualized and harvested following a 10-week exercise intervention. Figure 3B showed marked vessel congestion in the AS-SED and AS-EX groups. Here we presented representative H&E staining images of plaques observed in brachiocephalic arteries from three groups. Histological findings of plaque size and plaque stability were observed initially, the AS-EX group represented a smaller plaque size and increased plaque stability (Fig. 3B). In addition, en face aorta Oil Red O stained analysis revealed a 0.93-fold decrease in atherosclerotic lesions (Fig. 3A).

We performed Oil Red O staining and Masson staining on the aortic roots to measure the lesion areas in aortic root cross-sections (Fig. 3C). Oil Red O stains showed that the aortic plaque area had significantly increased whereas Masson stains showed a decrease in aortic collagen content in the AS-SED group compared with the Control group. Of great interest, the AS-EX group exhibited decreased plaque areas and increased collagen content (Fig. 3D, E). CD68 is an inflammation marker of macrophages. Immunohistochemical staining was performed for CD68. The results suggested that exercise reduced inflammatory infiltration (Fig. 3C, F). In addition, DHE staining also confirmed that exercise markedly reduced ROS level (Supplementary Fig. 1). These results further verified that exercise intervention reduced aorta lesion area and inhibited the aortic plaque formation but increased collagen in the plaque.

### Exosome isolation, miRNAs analysis and miRNA target gene

**Fig. 4:**
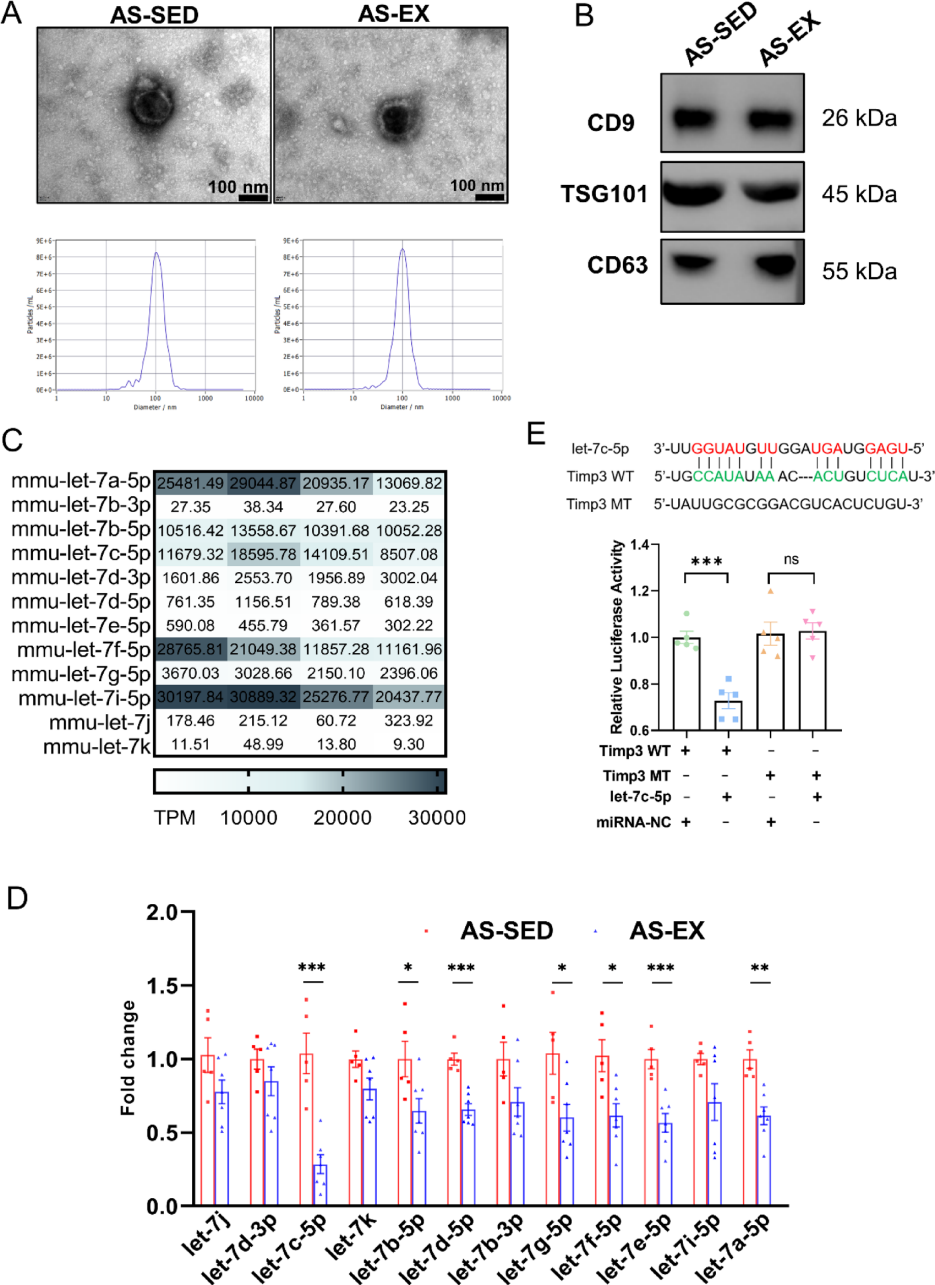
let-7c-5p negatively regulates Timp-3 expression. (**A**) Representative images of exosomes captured by transmission electron microscopy and NTA analysis showing the particle size distribution of serum-derived exosomes from two groups. (**B**) Exosome biomarkers include CD9, CD63, and TSG101 examined via Western blot analysis. (**C**) Results of the let-7 family expression by high-throughput sequencing. MiRNAs with TPM (Transcripts per million) values less than 1 were excluded. (**D**) Relative expression of let-7 family members in the aortas via qRT-PCR (n = 5–7 per group). The fold change was calculated based on delta Ct between endogenous U6 control. (**E**) Dual-luciferase assay to demonstrate the interaction between Timp-3 and let-7c-5p (n = 5 per group). *P < 0.05, **P < 0.01, ***P < 0.001, ns indicating *P* > 0.05.

Increasing evidence has indicated that miRNA regulatory networks are associated with AS progression^27^. Altered miRNA profiles in plasma exosomes after exercise intervention have been reported^28^. We next investigated whether exercise intervention effect was mediated by circulating exosome-derived miRNA transmission. Serum exosomes were isolated using ultracentrifugation. Electron micrographs of exosomes isolated from the AS-SED and AS-EX groups were obtained (Fig. 4A). NTA analysis indicated that the particle size distribution of exosomes was consistent with the size range of exosomes (average size 100 nm) (Fig. 4A). Furthermore, Western blot analysis confirmed the presence of characteristic exosomal markers, including CD9, CD63, and TSG101 (Fig. 4B). These data demonstrate the successful isolation of serum exosomes.

Numerous studies have implicated the aberrant expression of let-7 members in cardiovascular diseases, such as stroke, cardiac fibrosis, and AS as well as in the inflammation related to these diseases^29^. In the present study, Fig. 4C and Supplementary Fig. 2 presented the heatmap analysis of differentially expressed let-7 members between the AS-SED and AS-EX groups by miRNA sequencing. The relative expression levels of the let-7 family miRNAs were determined by qRT-PCR (Fig. 4D). In the AS-EX group, the expression of let-7c-5p, let-7d-5p, let-7e-5p, let-7a-5p, let-7b-5p, let-7g-5p, and let-7f-5p was significantly lower than those in the AS-SED group. Other members including let-7j, let-7d-3p, let-7 k, let-7b-3p, and let-7i-5p showed no significant differences between the two groups. Notably, let-7c-5p had the largest decrease. Therefore, we focused on let-7c-5p in subsequent studies.

To identify the downstream target of let-7c-5p, the present study used the online bioinformatics platform miRWalk to predict the potential target genes. The binding site of let-7c-5p to the target gene Timp-3 was shown in Fig. 4E. As for whether other members of the let-7 family share the same binding site, we conducted bioinformatics predictions and the results showed that other family members do not share this site (Supplementary Fig. 3). To verify the interaction between let-7c-5p and Timp-3, we constructed Timp-3 3′ UTR wild-type (WT) or mutant (MT) luciferase reporter plasmids for luciferase reporter assays. The results confirmed that let-7c-5p could directly complement the 3′UTR of the Timp-3, let-7c-5p inhibited the 3′UTR luciferase activity of Timp-3, while Timp-3 binding site mutations eliminated these inhibitory effects. These data demonstrate the interaction between Timp-3 and let-7c-5p.

### Treadmill exercise increases Timp-3 expression in vascular tissues

**Fig. 5:**
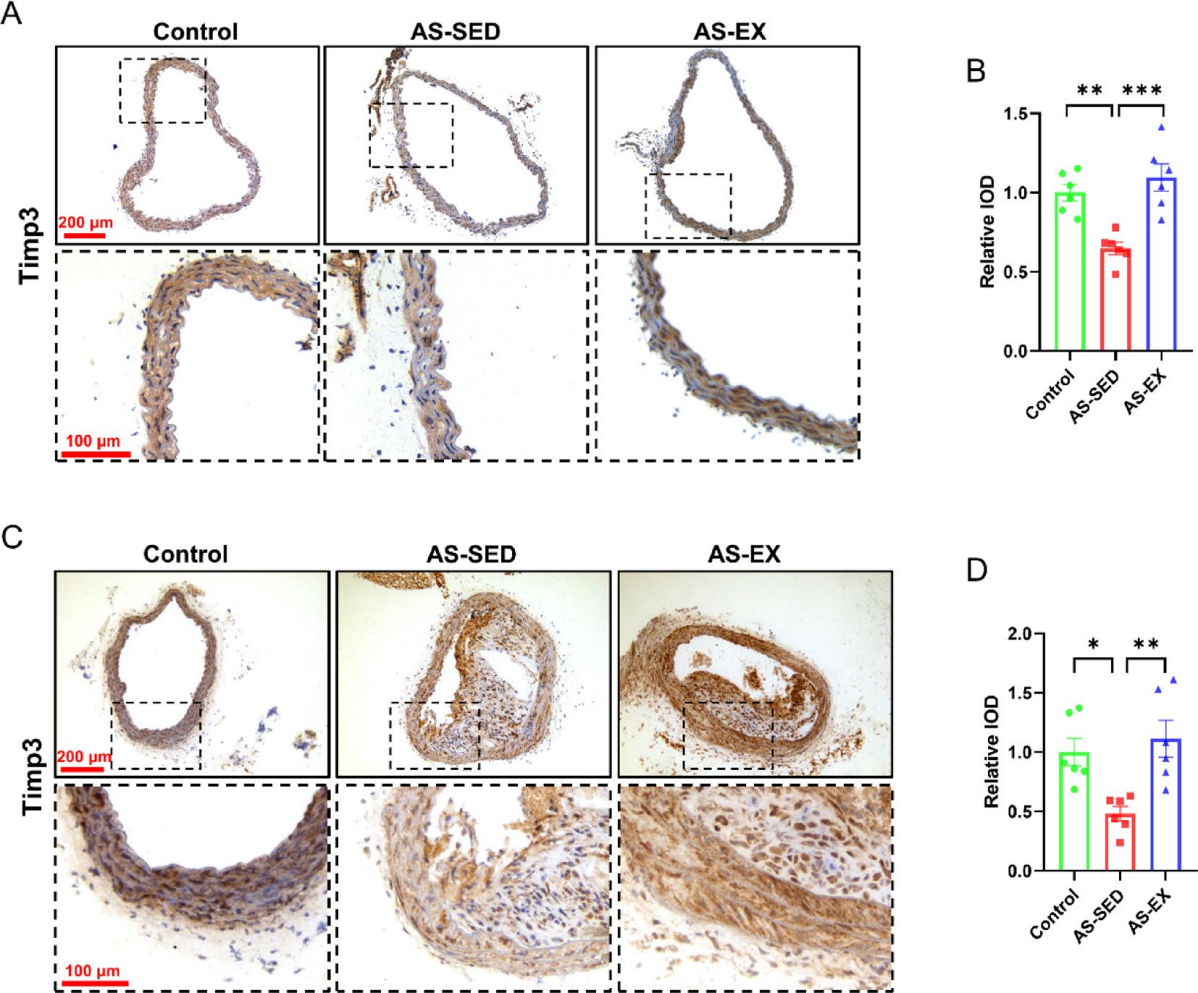
Exercise increases Timp-3 expression in vascular tissues. (**A**–**B**) Representative images and relative expression of Timp-3 in the thoracic aorta of mice from each group (n = 6 per group). (**C**–**D**) Representative images and relative expression of Timp-3 in the brachiocephalic artery of mice from each group (n = 6 per group). *P < 0.05, **P < 0.01, ***P < 0.001.

There are four proteins in the tissue inhibitors of metalloproteinases (Timps) family (Timp-1, Timp-2, Timp-3, and Timp-4) that serve as natural inhibitors of matrix metalloproteinases (MMPs) and play a vital role in maintaining the homeostasis of the ECM (Extracellular matrix)^30^. After exercise intervention, quantification of relative Timp-3 immunohistochemical staining in the thoracic aorta and brachiocephalic artery of mice was shown in Fig. 5. The results demonstrated that compared with the Control group, the expression of Timp-3 in both the thoracic aorta (Fig. 5A, B) and the brachiocephalic artery (Fig. 5C, D) decreased significantly in the AS-SED group, whereas the expression of Timp-3 was significantly increased in the AS-EX group. These data demonstrate an increase of Timp-3 in vascular tissues after exercise intervention.

### Treadmill exercise decreases the expression of MMP-9 and inflammatory factors in vascular tissues

**Fig. 6:**
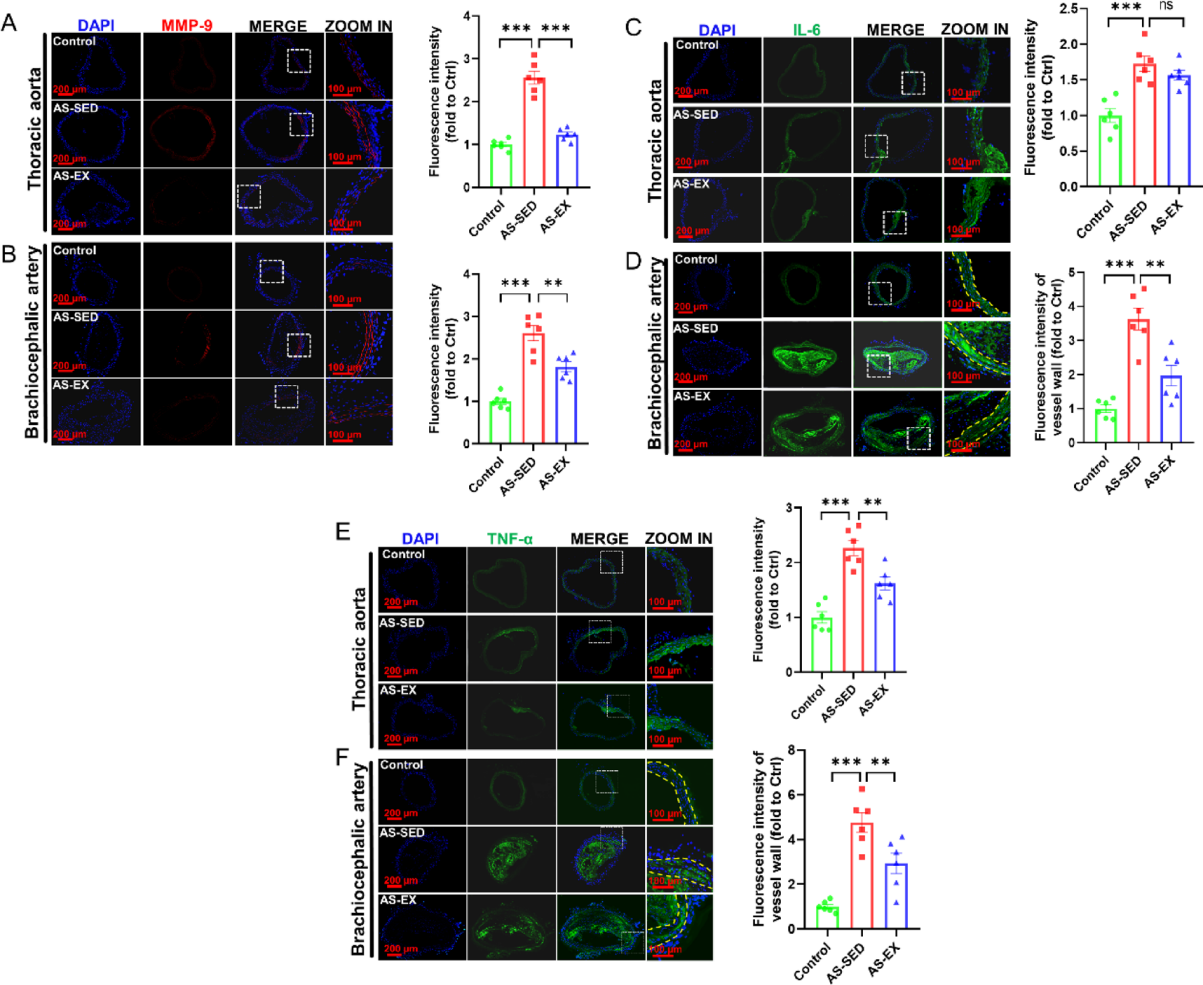
Exercise decreases the expression of MMP-9 and inflammatory factors in vascular tissues. (**A**–B) Immunofluorescence representative images and relative expression of MMP-9 (red) in the thoracic aorta and brachiocephalic artery, respectively (n = 6 per group). (**C**–**D**) Immunofluorescence representative images and relative expression of IL-6 (green) in the thoracic aorta and brachiocephalic artery, respectively (n = 6 per group). (**E**–**F**) Immunofluorescence representative images and relative expression of TNF-α (green) in the thoracic aorta and brachiocephalic artery, respectively (n = 6 per group). **P < 0.01, ***P < 0.001, ns indicating *P* > 0.05.

AS is characterized by persistent inflammation within the artery walls^31^. MMP-9 is closely associated with the degree of inflammation severity^32^. Hence, we tested the expression of MMP-9 and inflammatory factors containing IL-6 and TNF-α. The expression of MMP-9, IL-6, and TNF-α in the thoracic aorta and the brachiocephalic artery of mice was shown in Fig. 6. The immunofluorescence results revealed that compared with the Control group, the expression of MMP-9 (Fig. 6A, B), IL-6 (Fig. 6C, D), and TNF-α (Fig. 6E, F) increased significantly in both the thoracic aortas and the brachiocephalic arteries in the AS-SED group. Conversely, the AS-EX group showed the opposite results. The above results illustrate that treadmill exercise attenuates vascular inflammation.

### let-7c-5p regulates MMP9 expression and inflammatory factors via targeting Timp-3

**Fig. 7:**
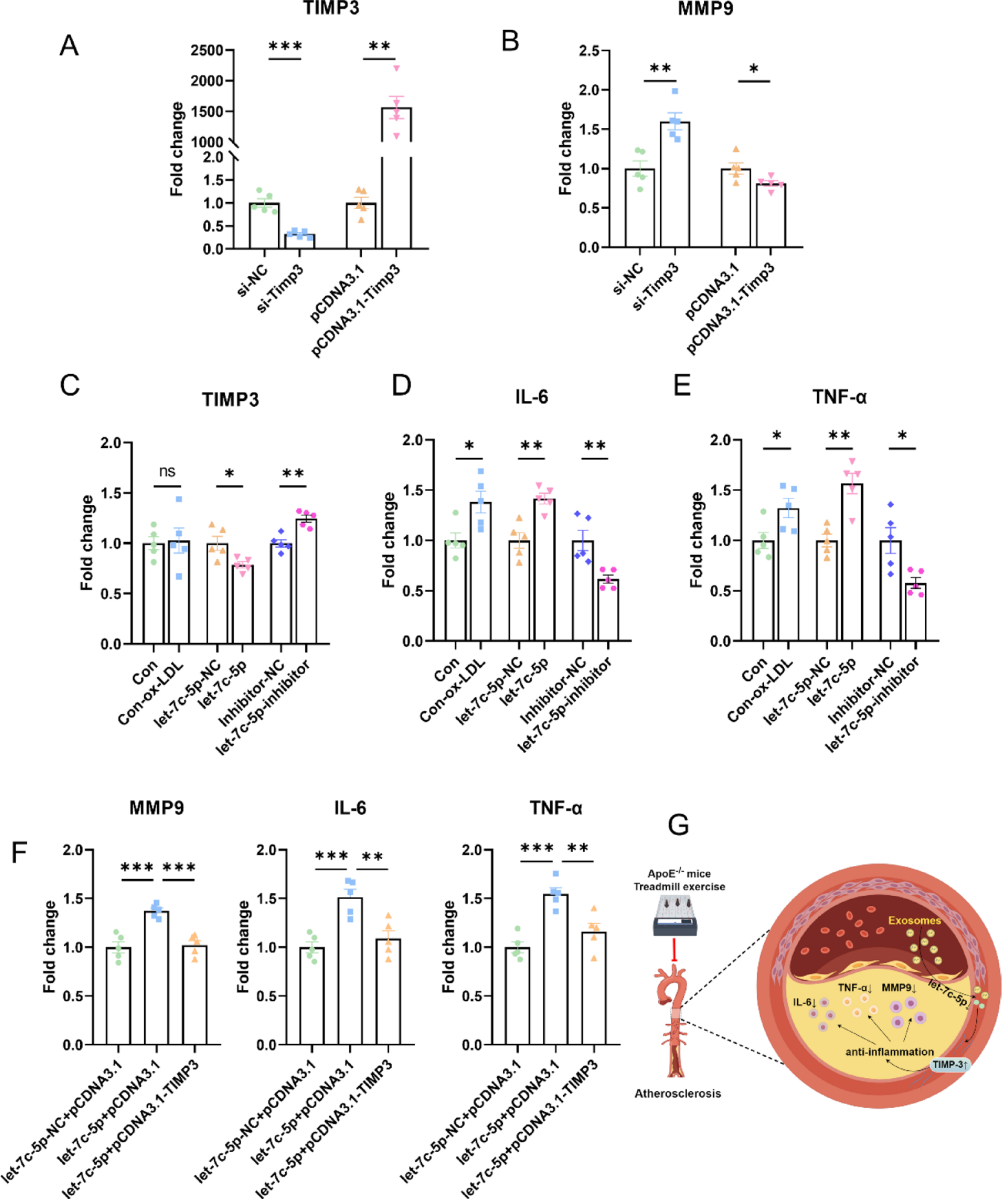
let-7c-5p ameliorates inflammation by regulating the expression of Timp-3. (**A**) The overexpression and knockdown efficiency of Timp-3 were verified by qRT-PCR analyses (n = 5 per group). si-Timp-3, siRNA for Timp-3 knockdown. pCDNA3.1-Timp-3 for Timp-3 overexpression. (**B**) MOVAS cells were transfected with si-Timp-3 or pCDNA3.1-Timp-3 and induced with ox-LDL, followed by qRT-PCR validation of MMP-9 expression (n = 5 per group). ox-LDL, oxidized low-density lipoprotein. (**C**–**E**) MOVAS cells were transfected with let-7c-5p mimics or let-7c-5p inhibitor and induced with ox-LDL, followed by qRT-PCR validation of Timp-3 (**C**), IL-6 (**D**), and TNF-α expression (**E**) (n = 5 per group). (**F**) Quantification of rescue experiments. (**G**) The schematic diagram illustrates the mechanism of treadmill exercise in stabilizing plaques and ameliorating atherogenesis. Treadmill exercise changes circulating exosomal miRNAs, reduces the level of let-7c-5p in serum and aorta, which increases the expression level of Timp-3, thus playing a role in alleviating atherosclerotic inflammation. The diagram was drawn by Figdraw (www.figdraw.com). *P < 0.05, **P < 0.01, ***P < 0.001, ns indicating *P* > 0.05.

Based on the preceding results, we further verified the results at the cellular level. Figure 7A depicts the change in Timp-3 and MMP-9 expression under si-NC, si-Timp-3, pCDNA3.1, and pCDNA3.1-Timp-3 transfection. Timp-3 expression was significantly reduced in si-Timp-3-transfected cells versus si-NC controls, while pCDNA3.1-Timp-3 transfection markedly increased Timp-3 levels compared with the empty pCDNA3.1 plasmid (Fig. 7A). Therefore, they could be used for subsequent experiments. VSMCs play a key role in various stages of AS^33^. Here we used the MOVAS cell (mouse smooth muscle cell line) subjected to ox-LDL induction for an in vitro model of cell injury. MMP-9 expression was elevated in si-Timp-3 cells relative to si-NC and reduced in pCDNA3.1-Timp-3 versus the pCDNA3.1 group, confirming that Timp-3 overexpression inhibits the expression of MMP-9 (Fig. 7B). Furthermore, we assessed the effect of let-7c-5p on the proliferation and oxidative stress of MOVAS. The results indicated that let-7c-5p inhibited cell proliferation and promoted ROS levels (Supplementary Fig. 4).

To further verify the regulation of inflammation by let-7c-5p through Timp-3, MOVAS cells were transfected with let-7c-5p mimics or let-7c-5p inhibitor, and the expression of Timp-3, IL-6, and TNF-α was assessed via qRT-PCR. Figure 7C presented that let-7c-5p inhibited the expression of Timp-3. While Timp-3 increased in the presence of let-7c-5p inhibitor. Furthermore, let-7c-5p promoted IL-6 and TNF-α expression, whereas transfection with the let-7c-5p inhibitor considerably decreased IL-6 and TNF-α expression (Fig. 7D, E). Next, we performed rescue experiments under Timp-3 overexpression in the presence of elevated let-7c-5p. The results indicated that overexpression of Timp-3 could inhibit MMP-9, IL-6 and TNF-α expression (Fig. 7F). These results suggest that let-7c-5p regulates inflammation by targeting Timp-3.

## Discussion

In the present study, we provided new evidence demonstrating the exercise effect in stabilizing atherosclerotic plaques, alleviating atherosclerotic lesions, attenuating inflammation and restoring endothelium-dependent vasodilatation to protect against AS in ApoE^−/−^ mice. We found that exercise resulted in lower circulating exosome-derived let-7c-5p levels. Moreover, let-7c-5p negatively regulated Timp-3 expression in the aorta. Suppressing let-7c-5p promotes the expression of Timp-3, thus inhibiting the expression of MMP-9, IL-6 and TNF-α in vitro cell model. Our data demonstrated that treadmill exercise ameliorates atherogenesis and vascular inflammation in ApoE^−/−^ mice via circulating exosome-derived let-7c-5p.

Previous studies suggest that a higher PWV is observed in ApoE^−/−^ mice with coronary atherogenesis^34,35^. Atherogenesis induces elevated cardiac afterload, which diminishes cardiac reserve capacity, highlighting impaired cardiac reserve capacity in the AS model^36^. Our data demonstrate that PWV was significantly decreased in AS mice subjected to exercise intervention, indicating that exercise attenuates atherogenesis. Echocardiographic analyses revealed that EF, FS and CO were significantly higher in AS-EX mice, confirming exercise-mediated mitigation of AS-induced cardiac dysfunction. Sirius red staining further demonstrated a significantly decreased fibrosis area in AS-EX mice compared with AS-SED mice, which was consistent with a prior study^37^, suggesting exercise alleviates AS-associated myocardial fibrosis and injury. Collectively, these findings confirm that treadmill exercise ameliorates PWV value, AS-associated cardiac dysfunction and myocardial injury.

Endothelial dysfunction is recognized as an early indicator of cardiovascular diseases. The endothelium, consisting of a monolayer of endothelial cells, is crucial for maintaining vascular homeostasis and function, and is involved in the initial stages of AS development^38^. Aortic ring data demonstrated that the AS-EX group displayed increased sensitivity to ACh and improved the impaired maximum endothelium-dependent vasodilation compared with the AS-SED group. This suggests that exercise intervention improved endothelium-dependent vasodilatation in AS mice. These improvements may arise from exercise-induced structural or functional adaptations, such as vascular smooth muscle remodeling or contractile protein modulation^39^. While endothelial contraction contributes to vascular homeostasis, our study prioritized relaxation function, due to its direct relevance to vascular protection—inhibiting VSMC proliferation, inflammation, oxidative stress, and thrombosis—particularly in plaque-prone regions with hemodynamic instability^40^. No significant differences were observed in the elastic fiber modulus and collagen fiber modulus among the three groups, potentially reflecting adaptive vascular wall remodeling to mechanical stress, such as collagen cross-linking or elastic fiber restructuring under pathological conditions^41^.

Treadmill exercise effectively reduces atherosclerotic plaque size while enhancing stability. We found a remarkable reduction in atherosclerotic plaque area in exercised ApoE^−/−^ mice. Oil Red O staining further demonstrated reduced plaque deposition of aortic root in the AS-EX group compared with the AS-SED group. Exercise reduces arterial lipid content, resulting in alterations in plasma lipid parameters. Studies have demonstrated that exercise intervention in AS leads to decreased plasma TC, LDL-C, and TG, along with increased HDL-C^42,43^. These changes contribute to reducing myocardial collagen deposition, suppressed apoptosis, improved cardiac function, and diminished oxidative stress, conferring significant benefits to both the heart and vasculature in AS^44–46^. Collagenis a critical determinant of fibrous cap mechanical strength and plaque stability^47^. Studies show that in the early stages of AS, the cross-linking structure of collagen molecules in the aorta of mice changes significantly. The arrangement and integrity of collagen fibers are damaged. Such damage can change the mechanical properties of the vascular wall and affect vascular stability^48^. In our study, Masson staining showed collagen was significantly increased in the aorta and plaques of the AS-EX group compared with the AS-SED group. These findings conclusively establish that exercise not only attenuates atherosclerotic plaque progression but also enhances plaque stabilization through collagen enrichment.

MiRNAs are the best known and most extensively studied of noncoding RNAs in the context of exercise. Exercise training dynamically regulates miRNAs, which are crucial for cardiovascular homeostasis^49^. In the present study, we isolated serum exosomes and performed miRNA sequencing, identifying differentially expressed miRNAs from the let-7 family. Among these, let-7c-5p was significantly differentially expressed with the largest fold change via qRT-PCR analysis. Among the many miRNAs expression differences detected, we focused on the let-7 family due to its well-documented high expression in the cardiovascular system and its established association with cardiovascular pathologies, including myocardial hypertrophy, myocardial fibrosis, dilated cardiomyopathy, arrhythmia, angiogenesis, AS, and hypertension^50^. Notably, recent studies have reported elevated circulating levels of let-7 in patients with hypertension and AS, further supporting its relevance to cardiovascular disease mechanisms^51^. We therefore selected let-7c-5p for further investigation.

According to the study by Panni et al., we utilized miRWalk for bioinformatic prediction of miRNA-mRNA interactions^52^. Timp-3 was identified as a potential target for let-7c-5p, which was validated by dual-luciferase assays. The immunofluorescence analysis further revealed elevated Timp-3 levels in both the thoracic aorta and brachiocephalic artery after exercise intervention. Timp-3 is a multifunctional regulator in the cardiovascular pathology, known to inhibit MMPs activity, modulate angiogenesis, suppress inflammation, regulate gene expression, and enhance cardiac function^53^. As a key member of the MMPs family, MMP-9 is closely linked to atherosclerotic plaque stability. MMP-9 deficiency attenuates aortic atherosclerotic burden by modulating macrophage infiltration and collagen deposition during disease progression, whereas its overexpression exacerbates ECM degradation, amplifies inflammatory responses, and promotes plaque destabilization^54^. Exercise training has been shown to stabilize atherosclerotic plaques by suppressing MMP activity, particularly MMP-9, which preserves collagen integrity—the primary ECM component—thereby reducing plaque size and enhancing stability^25,55^. Our study demonstrates a significant reduction in vascular MMP-9 expression following exercise intervention, the results were consistent with previous studies.

Inflammation is pivotal in AS progression, influencing the entire process from the initial recruitment of leukocytes to the rupture of unstable lesions^56^. Inflammatory mediators and cells critically regulate plaque formation, progression, and destabilization. Timp-3 binds to the ECM to inhibit MMP-2/9 activity and modulates cytokine release (e.g., TNF-α and IL-6), thereby suppressing inflammatory responses, cell migration, and proliferation. Conversely, Timp-3 deficiency promotes inflammatory monocyte polarization^57–59^. Prior studies have demonstrated that IL-6 affects collagen synthesis and plaque stability in murine AS models^60,61^. Our data revealed significantly reduced vascular IL-6 and TNF-α expression post-exercise, aligning with observed plaque stabilization in aortic root analyses. Collectively, these results indicate that exercise may upregulate Timp-3, suppress MMP-9 and attenuate inflammatory responses, thereby stabilizing AS plaques.

MOVAS cells-a murine aortic VSMC model—are widely used to study ox-LDL-induced vascular injury in AS^62,63^. While endothelial cells mediate early AS inflammation, MOVAS cells enable direct investigation of VSMC-ECM interactions in inflammatory microenvironments, circumventing the need for exogenous cytokine supplementation in endothelial models. Given VSMCs’ proximity to collagen-rich ECM, this cell model is particularly suited for validating Timp-3/MMP-9 interactions. Atherosclerotic VSMCs exhibit elevated secretory markers (e.g., OPN, MMPs) compared with healthy counterparts^64^. Consistent with this, the qRT-PCR analysis revealed an increase in MMP-9 expression following Timp-3 silencing, and a decrease in MMP-9 expression following Timp-3 overexpression, confirming Timp-3-mediated MMP-9 regulation and supporting our mechanistic conclusions.

Ultimately, the functional importance of let-7c-5p was experimentally confirmed using miRNA mimics and miRNA inhibitor. Multiple miRNAs have been reported to modulate Timp-3^65,66^, this study provides new evidence of the interaction between let-7c-5p and Timp-3. Our data showed that let-7c-5p promoted IL-6 and TNF-α expression with a decline in Timp-3, whereas transfection with the let-7c-5p inhibitor considerably decreased IL-6 and TNF-α expression with a rise in Timp-3. Together, these results suggested that let-7c-5p suppresses MMP-9 expression and inflammatory factor production through Timp-3 downregulation. As one of the earliest identified miRNA families, let-7 is ubiquitously expressed across physiological systems, including the cardiovascular system, and its dysregulation is implicated in diverse pathologies^29,67^. Based on these research, we propose that exercise ameliorates AS by reducing let-7c-5p levels in serum, thereby increasing Timp-3 expression in VSMCs. This cascade suppresses MMP-9 expression and reduces levels of inflammatory factors. The reduction in inflammatory factors enables VSMCs to participate in normal collagen synthesis, thereby stabilizing plaques and ultimately ameliorating AS (Fig. 7G). The translational potential of inhibiting let-7c-5p in AS represents a promising avenue for research, particularly given its involvement in lipid metabolism and endothelial apoptosis. Inhibition of let-7c has been demonstrated to attenuate the progression of AS and reduce macrophage lipid accumulation by enhancing the PGC-1α/LXRα/ABCA1/G1 pathway, which is essential for maintaining cholesterol homeostasis in macrophages^68^. This evidence suggests that targeting let-7c may serve as a viable strategy for modulating lipid metabolism in atherosclerotic conditions. Furthermore, miRNA let-7c has been implicated in the regulation of endothelial cell apoptosis and dysfunction, which are critical events in the pathogenesis of AS^69^. The potential of let-7c-5p as a biomarker for cardiovascular diseases, including AS, is also noteworthy. Plasma-derived exosomal let-7c-5p has emerged as a promising biomarker for stable coronary artery disease, underscoring its diagnostic potential^70^. In conclusion, the inhibition of let-7c-5p represents a comprehensive strategy for addressing AS by simultaneously targeting lipid metabolism and endothelial dysfunction. This approach holds significant translational potential for the development of novel therapeutic strategies aimed at treating AS and associated cardiovascular diseases.

We additionally conducted a comparative analysis of the role of let-7c-5p in relation to other miRNAs previously associated with physical activity and AS, specifically miR-126, miR-146a, miR-155, and miR-492. Although these miRNAs are involved in the adaptive response to exercise, their primary functions appear to be distinct yet potentially complementary to that of let-7c-5p. For example, exercise interventions may impede the progression of AS by upregulating miR-492 and downregulating resistin within the aortic endothelium. Such intervention has been shown to modulate glucose and lipid metabolism, alleviate endothelial insulin resistance, and repair endothelial injury^19^. Additionally, in both exercise interventions and statin treatments in atherosclerotic mice, there was an observed increase in miR-146a and miR-126 levels within blood vessels, while miR-155 levels decreased. Notably, miR-146a interacts with the 3’ untranslated region of the TRAF6 gene, thereby reducing vascular TRAF6 and TLR4 signaling and mitigating vascular inflammatory damage in AS^18^. In contrast, our findings confirmed the regulation of Timp-3 by let-7c-5p, which subsequently influences the inflammatory response. Therefore, we propose that let-7c-5p may serve as a complementary approach within the intricate network of exercise-induced benefits.

This study underscores the therapeutic advantages of treadmill exercise in addressing AS. But several limitations warrant consideration. Primarily, the study’s exclusive focus on male mice represents a significant limitation. Although this approach is common in experimental design, it is crucial to recognize that biological sex is a critical factor influencing cardiovascular disease and treatment outcomes. Future research should be explicitly designed to explore these mechanisms in female models to ascertain potential sex-specific effects and to provide a more comprehensive understanding of the impact of exercise on AS. Second, studies that elucidate the molecular mechanisms underlying the regulation of Timp-3 expression mediated by let-7c-5p through in vivo rescue experiments could provide critical insights into the pathogenesis of AS progression. Mechanistically, although we have identified the involvement of let-7c-5p, its interactions with other regulatory networks, particularly those related to lipid metabolism and oxidative stress, remain incompletely understood. Furthermore, our inflammatory profiling primarily focus on IL-6 and TNF-α, thereby neglecting other significant mediators such as IL-1β and chemokines, which may play crucial roles in exercise-induced immunomodulation. These gaps highlight the necessity for more comprehensive pathway analyses in future research endeavors.

## Conclusion

In conclusion, our findings indicate that treadmill exercise mitigates atherogenesis and vascular inflammation through regulation of let-7c-5p. This protective mechanism is largely due to the exercise-induced downregulation of circulating exosome-derived let-7c-5p, which leads to decreased let-7c-5p and increased Timp-3 expression in vascular tissues. The elevated Timp-3 inhibits MMP-9 expression, thereby reducing collagen degradation and facilitating the development of more stable atherosclerotic plaques. Additionally, exercise downregulates MMP-9 expression and decreases levels of pro-inflammatory factors such as IL-6 and TNF-α, thereby attenuating plaque progression and enhancing overall plaque stability.

## Supplementary Information

Below is the link to the electronic supplementary material.

Supplementary Material 1

Supplementary Material 2

Supplementary Material 3

Supplementary Material 4

Supplementary Material 5

## Data Availability

The datasets used or analyzed during the current study are available online. RNA-seq data have been deposited at NCBI BioProject. The raw data have been deposited under the accession number: PRJNA1193121.

## Abbreviations

ACh: Acetylcholine
ApoE: Apolipoprotein E
AS: Atherosclerosis
CEM: Collagen elastic modulus
CO: Cardiac output
ECM: Extracellular matrix
EEM: Elastin elastic modulus
EF: Ejection fraction
FS: Fractional shortening
H&E: Hematoxylin-eosin
IL-6: Interleukin-6
miRNA: MicroRNA
MMP: Matrix metalloproteinase
MOVAS: Mouse aortic smooth muscle cells
NTA: Nanoparticle tracking analysis
ox-LDL: Oxidized low-density lipoprotein
PWV: Pulse wave velocity
qRT-PCR: Quantitative real-time polymerase chain reaction
siRNA: Small interfering RNA
TEM: Transmission electron microscope
Timp: Tissue inhibitor of metalloproteinase
TNF-α: Tumor necrosis factor-α
VSMCs: Vascular smooth muscle cells
