# Supplementary figures and images for: Treadmill exercise ameliorates atherogenesis and vascular inflammation in ApoE−/− mice via circulating exosome-derived let-7c-5p
Source: Sci Rep. 2025 Dec 9;16:585. doi: 10.1038/s41598-025-30174-3 (PMC12775415; doi:10.1038/s41598-025-30174-3)

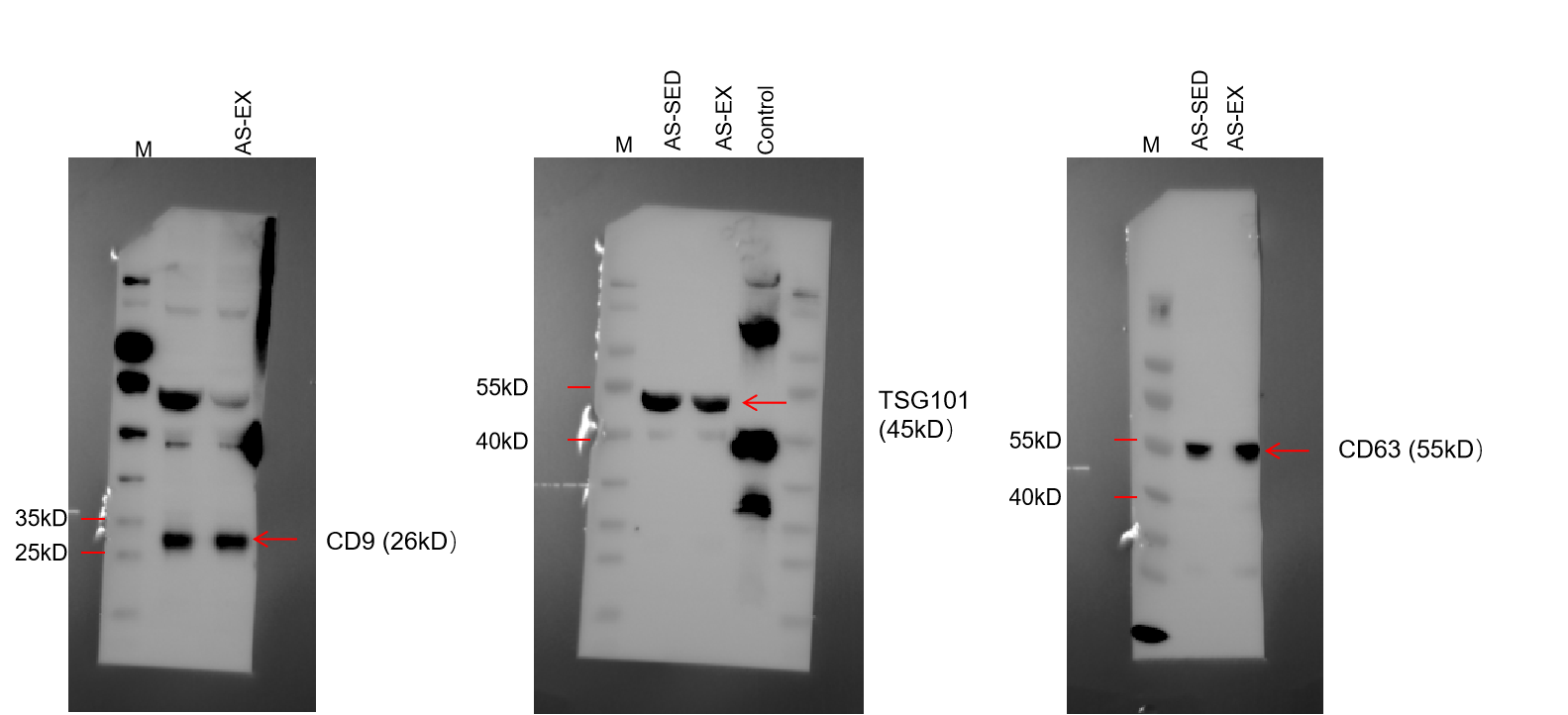

Supplement: Supplementary file 1 — Supplementary Material 1 [file 41598_2025_30174_MOESM1_ESM.tif]
