# Supplementary material for: Treadmill exercise ameliorates atherogenesis and vascular inflammation in ApoE−/− mice via circulating exosome-derived let-7c-5p
Source: Sci Rep. 2025 Dec 9;16:585. doi: 10.1038/s41598-025-30174-3 (PMC12775415; doi:10.1038/s41598-025-30174-3)
Supplement: Supplementary file 4 — Supplementary Material 4 [file 41598_2025_30174_MOESM4_ESM.pdf]

Supplementary information

Treadmill exercise ameliorates atherogenesis and vascular inflammation in ApoE$^{-/-}$ mice via circulating exosome-derived let-7c-5p

Wenhuang Guo, Jinyun Wang, Zaoshang Chang, Shuo Lin, Guangyuan Sha, Shen Wang, Junhao Huang, Min Hu, Jingbo Xia

Supplementary Figure 1. Exercise reduced ROS level in aortic root cross-sections.
Supplementary Figure 2. Heatmap of the 215 miRNAs profiled.
Supplementary Figure 3. Bioinformatics predictions of the binding relationship between let-7 family members and Timp3.
Supplementary Figure 4. The effect of let-7c-5p on proliferation and oxidative stress of MOVAS.

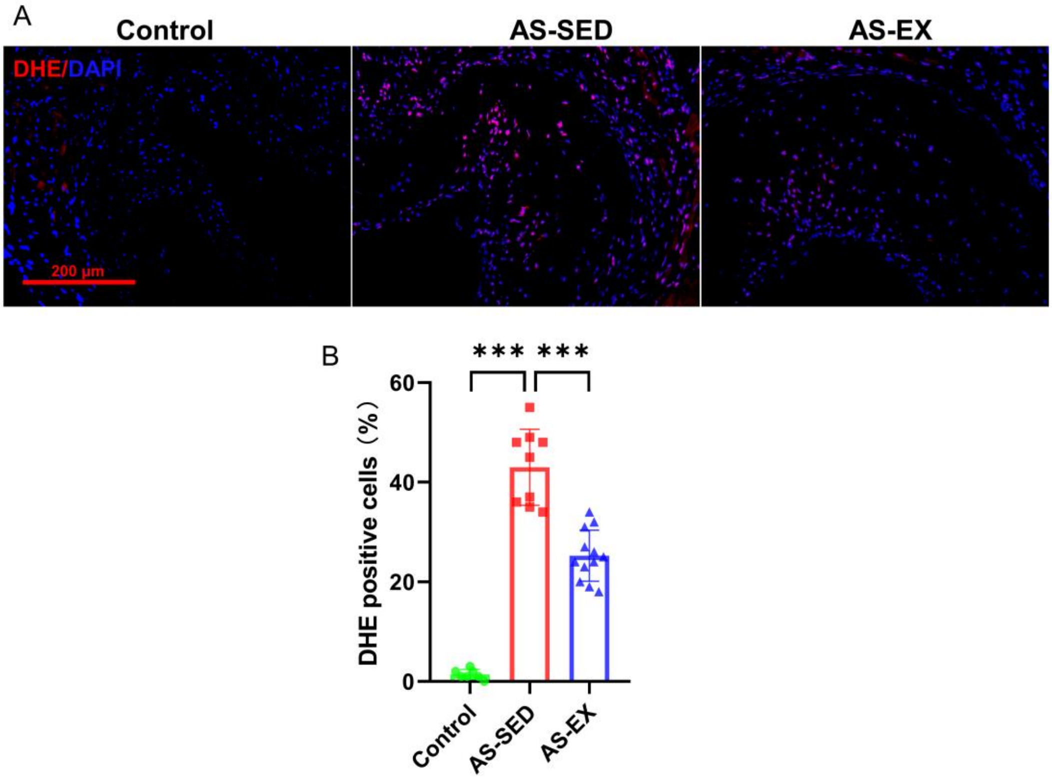

Supplementary Figure 1. Exercise reduced ROS level in aortic root cross-sections. (A) Representative images of DHE staining. (B) Quantification of the DHE positive cells (n=7-12 per group). ***P < 0.001.

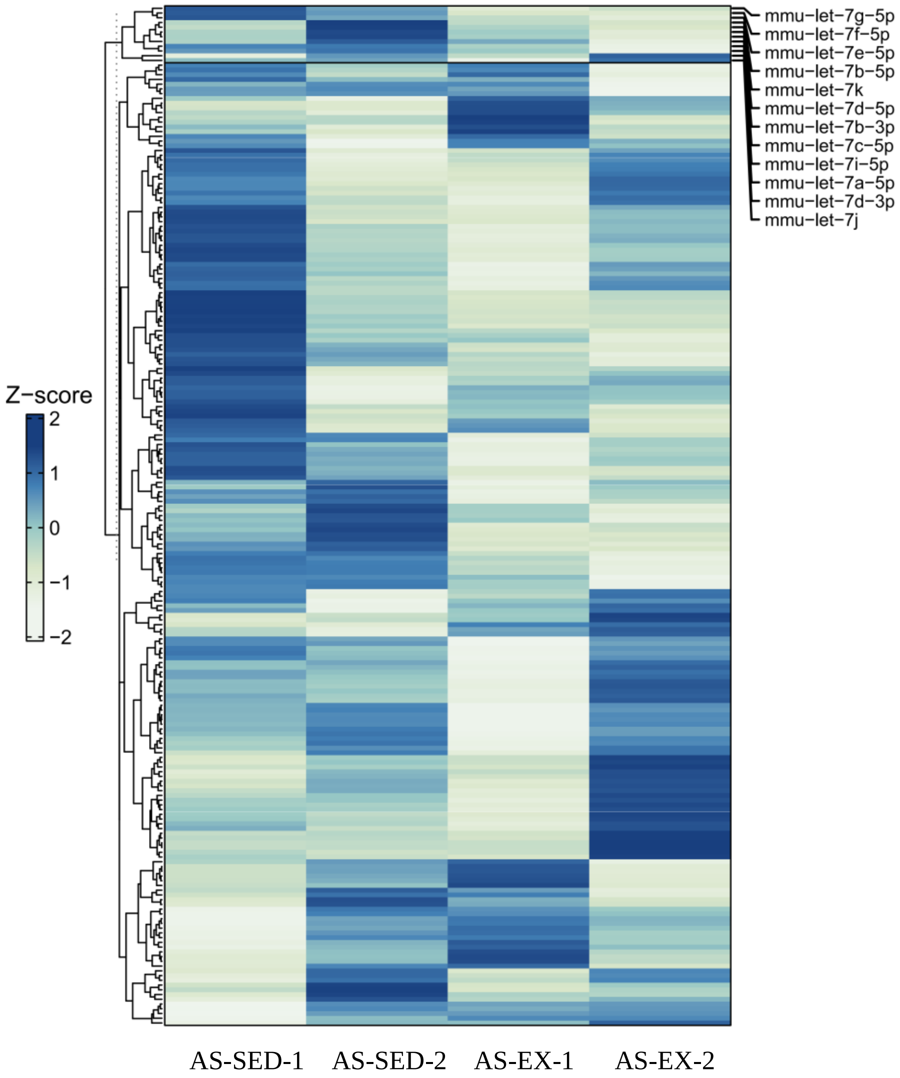

Supplementary Figure 2. Heatmap of the 215 miRNAs profiled. The transformed TPM value log2 (TPM+1) were visualized using the heatmap. By performing pseudocounting and log10 conversion on each value in the matrix, followed by Z-score normalization at the miRNA level.

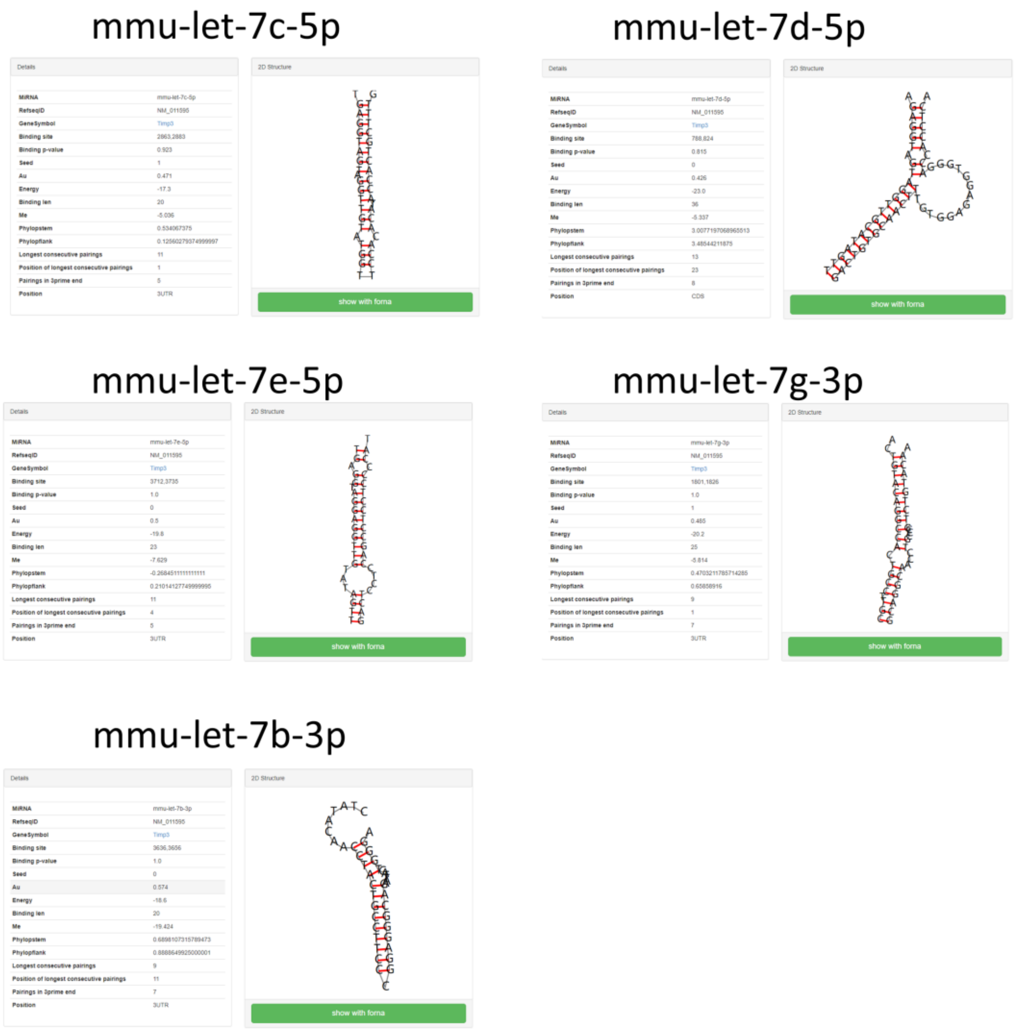

Supplementary Figure 3. Bioinformatics predictions of the binding relationship between let-7 family members and Timp3. According to bioinformatics predictions, a total of 5 family members, including let-7c-5p, can bind but do not share the same binding site.

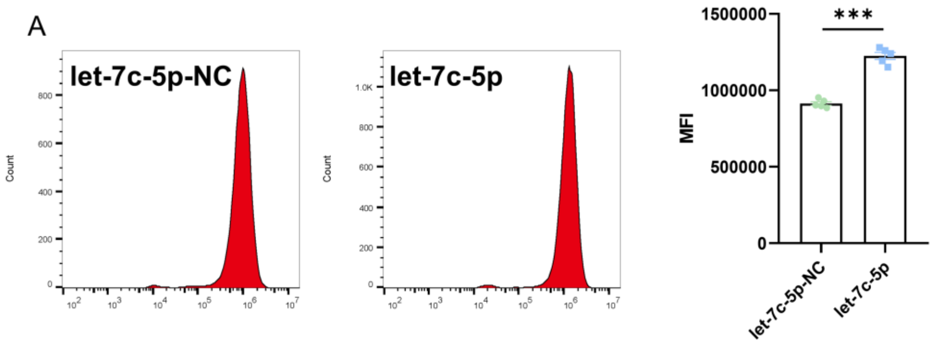

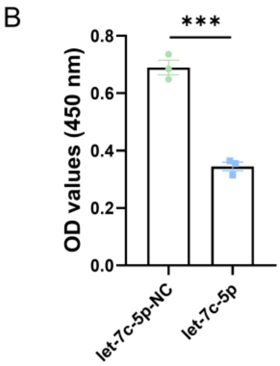

Supplementary Figure 4. The effect of let-7c-5p on proliferation and oxidative stress of MOVAS. (A) The intracellular ROS generation was evaluated by measuring the oxidation CM-H2DCFDA. (B) Cell proliferation was measured through CCK8 assays. ***$P < 0.001$.
